# Effectiveness and Experiences of Online Mental Health Peer Support for Young People: Systematic Scoping Review

**DOI:** 10.2196/83139

**Published:** 2026-02-25

**Authors:** Shuting Yuan, Gavin Davidson, Sebastian Kurten, Paul Best

**Affiliations:** 1 School of Social Sciences, Education and Social Work, Queen's University Belfast, University Road, Belfast, BT7 1NN, United Kingdom, 44 28 9097 3151; 2 Department of Interdisciplinary Social Sciences, Utrecht University, Utrecht, The Netherlands

**Keywords:** online peer support, youth mental health, digital interventions, virtual communities, social media

## Abstract

**Background:**

The prevalence of mental health conditions among young people is high and further increasing. Despite this considerable need, barriers remain to accessing and engaging with traditional mental health services. Online mental health peer support is increasingly popular among young people seeking help. However, research examining the effectiveness of online mental health peer support and user-centered experiences remains limited.

**Objective:**

This systematic scoping review aimed to synthesize research evidence on the effectiveness and experiences of online mental health peer support for young people, compare these across different forms, and identify possible applications of online peer support.

**Methods:**

This scoping review followed the 5-stage framework proposed by Arksey and O’Malley and revised by Levac et al. Three reviewers screened the articles. The IBSS, SSCI, Scopus, PsycINFO, MEDLINE, and Social Policy and Practice databases were searched by title and abstract. Retrieved studies (N=8327) were double-screened, and 38 articles met the inclusion criteria. Studies were included if they focused on young people aged up to and including 25 years and if the intervention was online peer support primarily aimed at supporting mental health.

**Results:**

The number of participants (posts/comments) in each study ranged from 10 to 36,934. Seventeen studies reported on the effectiveness of online peer support, and 28 studies reported on young users’ experiences. This review summarized evidence of overall positive clinical outcomes, personal recovery outcomes (including improved social connectedness and other personal recovery outcomes), and multidimensional experiences of online mental health peer support (such as fostering resonance or fatigue).

**Conclusions:**

Overall, online mental health peer support demonstrated positive effects on clinical and personal recovery outcomes. However, findings related to user experiences were mixed. Experiences were influenced by factors such as safety, anonymity, and the quality of peer interactions. These insights may inform the role alongside traditional services, attractive platform design, and safeguarding. Future research should further explore the integration of online peer support with traditional services and various digital platforms to better address young people’s mental health needs and further examine its effectiveness as well as experiences in practice to maximize the peer support benefits and reduce risks.

## Introduction

Mental health is a matter of global concern within the domain of public health. In recent years, young people have faced increasing risk of mental health challenges [1,2]. The United Nations Children’s Fund [3] reported that 13% of young people globally lived with a diagnosed mental health condition. Similarly, official statistics from England show that the proportion of probable mental disorders among young people aged 17‐19 and 20‐25 years was as high as 23.3% and 21.7%, respectively [4]. This trend may reflect both rising prevalence and increased awareness, detection, and service demand. Regardless of the relative contribution of these factors, the need for mental health interventions for young people is growing [5].

Although young people experience mental health conditions at higher rates, they often hesitate to seek help from conventional services because of stigma or a desire to go it alone [6]. Young people with mental health issues are more likely to seek support from others facing similar concerns [7]. Therefore, peer support is increasingly recognized as an important element of mental health services [8]. Here, “peers” refers to individuals who share experiences of mental distress [9], and peer support can be defined as “the process of providing and receiving assistance between individuals who share characteristics or lived experiences” (p. 1120) [10]. Peer support is based on the fundamental principles of respect, shared responsibility, and mutual agreement [11]. Initially developed offline, peer support for youth mental health has been shown to produce positive outcomes. A systematic review of five studies on offline peer support found benefits, including reduced anxiety, increased self-esteem, and higher perceived social support [12]. Halsall et al [13] reported that offline peer support can enhance hope and promote empowerment. Meanwhile, Conley et al [14] proposed that offline peer support may reduce mental health stigma, enhance stigma-related coping mechanisms, and boost disclosure-related self-efficacy. These effects may, in turn, increase the likelihood that young individuals access additional services and support [14]. A recent scoping review also demonstrated that offline peer support was associated with fewer barriers for young adults accessing mental health services compared with traditional care [15]. It is evident that offline peer support appears to be a promising and relatively accessible pathway toward improving young people’s mental health [16], and it is gradually becoming embedded in national mental health policy in many countries [9,17].

Despite the fact that offline peer support is undoubtedly beneficial to youth mental health, there is an apparent decline in interest among young people primarily due to the following reasons. The rapid expansion of digital mental health services has fundamentally transformed how mental health care is delivered, prompting a significant shift in research priorities toward online mental health peer support [11]. Reflecting this transformation, the National Health Service in the United Kingdom has committed increased financial investment to the development and integration of digital platforms and online support systems as part of its long-term strategic plan [18]. The COVID-19 pandemic has further increased the provision and use of online peer support interventions, with the aim of equipping young people with necessary coping skills [10] and enhancing their overall well-being [19]. Furthermore, social media platforms are frequently employed by young individuals to interact with others grappling with similar mental health challenges [20]. Apart from external factors, young people actively turn to online space for obtaining mental health support. A mounting body of evidence suggests that online interventions are particularly appealing to young people with mental health issues [20-23]. Research indicates that young people as “digital natives” frequently use the internet to seek mental health support and report high levels of satisfaction with online resources [21,24,25].

Consequently, online peer support has the potential to complement existing mental health services facing high demand and unmet needs. However, evidence on its effectiveness remains mixed. For example, in a systematic review of online peer support for youth mental health, Ali et al [26] found positive effects in 2 out of 6 randomized controlled trials. While some studies link online peer support with greater compassion, improved clinical outcomes, and new social connections [27,28], others highlight potential risks, such as distress from reading mental health–related online content [29] and the possibility of social contagion [30]. The online environment offers benefits such as shared experience and anonymity; it also poses challenges, including problematic interactions and non–evidence-based advice.

In recent years, various forms of online mental health peer support have emerged; however, the relative benefits and drawbacks of these formats remain unclear. This systematic scoping review, therefore, aims to synthesize evidence on the effectiveness and experiences of online mental health peer support for young people, compare these across different forms, and identify possible applications of online peer support. Specifically, this review addresses the following questions:

1. What evidence has been reported in the literature regarding the effectiveness and experiences of online peer support?
2. Do effectiveness or experiences vary by type of online peer support?
3. What are the implications of these findings for the development of online peer support?

## Methods

### Overview

This systematic scoping review was conducted using the 5-stage framework proposed by Arksey and O’Malley [31] and revised by Levac et al [32]. This framework comprises the following stages: identifying the research question; identifying relevant studies; study selection; charting the data; and collating; and then summarizing and reporting the results. The review was also reported in accordance with the PRISMA-ScR (Preferred Reporting Items for Systematic Reviews and Meta-Analyses extension for Scoping Reviews; Checklist 1).

### Search Strategy

The objective of this review was to identify studies examining the effectiveness and experiences of online peer support for young people experiencing mental health difficulties. Two authors (SY and GD) drafted, developed, and implemented the search strategy in consultation with an experienced university librarian, who provided suggestions on search strings and database selection. There was unanimous consensus among all authors on the search strategy. A preliminary search was conducted in 6 databases: IBSS, SSCI, Scopus, PsycINFO, MEDLINE, and Social Policy and Practice databases using title and abstract in January 2024. Each database used equivalent searches. To ensure the inclusion of up-to-date articles, a second search was conducted in February 2025, employing the identical search strategy. The search did not use any database limits related to date in order to gain a comprehensive overview. The terms employed were predominantly derived from the realm of online delivery, peer support, mental health challenges, and young people in peer-reviewed articles. The following search terms were used:

- online OR virtual OR internet OR web-based OR digital
- AND “peer support” OR “support group*” OR communit* OR forum* OR chatroom*
- AND “mental health” OR “mental disorder*” OR “mental distress” OR “mental illness*” OR psycho* OR depression OR anxiet* OR personalit*
- AND child* OR “young people” OR “young adult*” OR “young person*” OR teen* OR adolescen* OR youth

### Eligibility Criteria

Inclusion and exclusion criteria were established in accordance with the population, concept, and context model as outlined in the Joanna Briggs Institute’s Manual for Evidence Synthesis [33] and are presented in Multimedia Appendix 1. In terms of population, the focus was on young people engaging in online peer support, but there was no age restriction used for people who were involved in providing, facilitating, and/or moderating this support. Reference lists of all included studies were hand-searched to identify eligible studies.

### Evidence Selection

The first round of search conducted in January 2024 yielded a total of 7591 articles, which were subsequently imported into EndNote with the objective of identifying instances of duplication. Subsequently, the remaining records (n=5215) were imported into Covidence, a tool designed for the management of screening [34], in order to conduct a second removal of duplication and select potential studies that were relevant to the review. Prior to the screening process, a consensus was reached among all authors concerning a set of inclusion and exclusion criteria. Following the second duplication removal process, the titles and abstracts of the remaining 4883 papers were double-screened. One reviewer (SY) screened all the potential studies, while the other 2 reviewers (GD, PB) each screened 50%. A total of 89 articles were retained to full-text double screening by the same 3 reviewers, who had completed the initial screening. Any discrepancies between the 2 perspectives were resolved by a third reviewer or through collaborative meetings. Following a thorough examination of the full texts, 59 papers were deemed to be ineligible and consequently excluded from the study. The second search, conducted in February 2025, yielded 736 records in total. The process of screening for articles retrieved in the second round is identical to the process employed in the first round. Following the removal of 386 duplicates (via EndNote, n=384; Covidence, n=2), 350 papers were screened for eligibility. After the initial screening of titles and abstracts, 331 articles were excluded. This process was followed by a full-text screening, which resulted in the exclusion of a further 12 articles.

### Data Extraction and Coding

Following deliberations within the team, a standardized template was created in Microsoft Excel spreadsheet, which was then used by one author to extract data from the included papers. It is important to note that one author reported the latest data extraction as well as information that was difficult to make a decision on to other authors in time and all authors convened at regular intervals, either in person or via online platforms, with the objective of ensuring consistency throughout the data extraction phase. The coding scheme was used to summarize the relevant components of online mental health peer support and explore the research questions. In addition to the effectiveness of online peer support, personal experiences of young people offer further insights into the benefits of this approach and provide a more detailed explanation of the underlying reasons for the observed outcomes. The coding scheme included author (year), country, data source, intervention based on three main characteristics (interaction, moderation, and targeted mental health conditions), effectiveness (clinical outcomes, personal recovery outcomes, and potential challenges), and experiences (positive or negative) of online peer support.

## Results

### Study Characteristics

**Figure 1.**
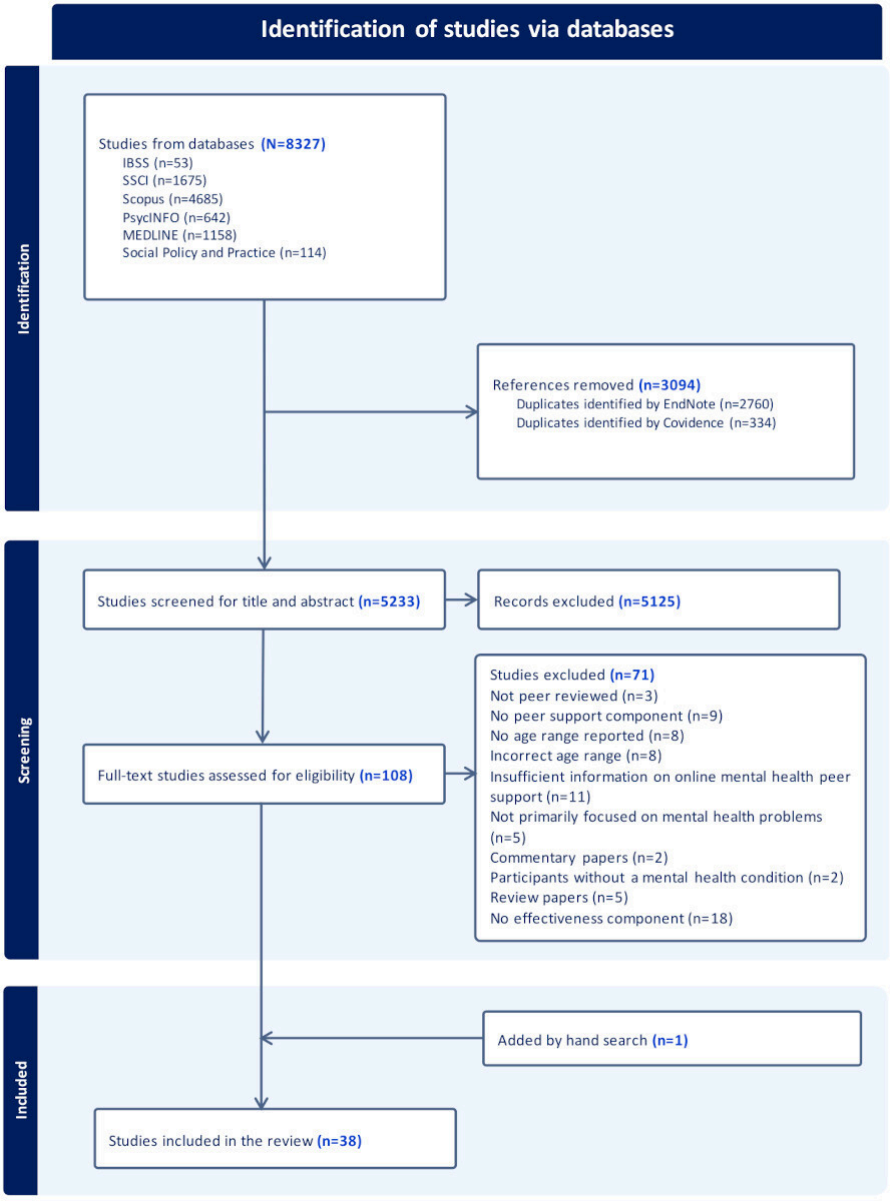
PRISMA-ScR flow diagram summarizing the evidence selection process. PRISMA-ScR: Preferred Reporting Items for Systematic Reviews and Meta-Analyses extension for Scoping Reviews.

The combined study selection process across both search rounds is illustrated in Figure 1, along with detailed reasons for exclusion. A total of 38 studies were included in the final review. Detailed characteristics of the included papers (n=38) are presented in Multimedia Appendix 2 [2,10,19,23,28,35-46] and Multimedia Appendix 3 [10,19,20,23,30,38,43,44,46-65], which categorize the studies according to their focus on the effectiveness and experiences of online mental health peer support. Of the 38 studies, 30 were published after 2019. Half of the interventions (n=19) did not report the country in which they were conducted. The number of participants (posts/comments) in each study ranged from 10 to 36,934. Participants were recruited offline in the United Kingdom (n=4), the United States (n=5), Australia (n=5), China (n=1), Singapore (n=1), Korea (n=1), Indonesia (n=1), and South Africa (n=1). The source of participants was categorized as specific clinics (n=6), schools (n=9), and online platforms (n=26). Most studies reported experiences of online peer support (n=28), with 17 studies reporting outcomes. Seven studies provided findings on both effectiveness and experiences.

### Intervention

The main characteristics of the interventions were categorized as follows: (1) synchronous or asynchronous interaction, (2) moderated or unmoderated delivery, and (3) general or specific mental health conditions targeted. This categorization was based on a scoping review identifying typologies of online mental health peer support [66]. When reported, the majority of online peer support interventions were asynchronous (n=31). A combination of synchronicity and asynchronicity was also reported in some studies (n=4). Only 2 studies specifically mentioned synchronous interaction with peers; in other studies, it was not specified, and so it was difficult to determine whether the interaction was synchronous or asynchronous. Two studies did not provide any details concerning the interaction. With regard to the issue of moderation, roles were divided into the following categories: professionals (n=12), trained and paid staff or workers (n=2), trained and paid peer support workers or peer moderators (n=3), peer support experts or specialists (n=2), trained peer volunteers (n=1), trained lay counselors (n=1), administrative moderators (n=1), researchers (n=2), unpaid peer moderators (n=1) and consumers (n=2). Furthermore, online peer support had the potential to address both general (n=26) and specific (n=11) mental health conditions.

### Outcome Measures

#### Classification

Effectiveness outcomes were organized into 2 main categories: clinical outcomes and personal recovery outcomes [67,68]. Clinical recovery focuses on an individual’s psychiatric symptoms and functioning, whereas personal recovery emphasizes broader social outcomes, including adaptation to illness, establishing an identity apart from the illness, and finding meaning, purpose, and hope in an individual’s journey of growth and personal development [68]. Some outcomes, such as engagement, attrition, adherence, and cost-effectiveness, could be considered in either category; however, for the purpose of this review, they were considered as an aspect of personal recovery. Personal recovery outcomes were further informed by the CHIME framework, which includes Connectedness, Hope and optimism, Identity, Meaning and purpose, and Empowerment [69], which covers both social interactions with peers and intrinsic personal enhancement. To circumvent any potential overlaps and to present the outcomes of the online peer support in as comprehensive a manner as possible, clinical outcomes (with a focus on mental health symptoms or the degree of psychiatric symptomatology) and personal recovery outcomes adapted from CHIME (connectedness and other personal recovery outcomes) were employed to measure the effectiveness of online peer support in this review.

#### Clinical Outcomes

The review showed a range of positive effects on clinical outcomes. A decline in various clinical symptoms, particularly with the involvement of professionals and rigorous moderation, was observed, including depressive symptoms, depression, anxiety, suicidal ideation, nonsuicidal self-injury (NSSI), self-harm, psychological distress, and negative emotional symptoms [2,10,23,28,37,40,41,44,46], thus improving mental well-being [10,19,46]. Morris and his colleague [36] reported that an online peer support platform, therapeutically supported by cognitive behavioral therapy, could reduce depressive symptoms, particularly among individuals who made repeated use of the service. Similarly, online peer support and cognitive behavioral therapy, as 2 components of online intervention, yielded substantial clinical benefits (eg, reduced depression and anxiety) for young individuals grappling with mental health challenges [46]. Concurrently, the provision of online peer support, either as a standalone intervention or in conjunction with online counseling, yielded substantial benefits in terms of enhanced clinical outcomes [44]. It also helped assist in the mitigating adverse sentiments toward medicine and illness, thereby enhancing engagement with mental health treatment [35]. There was also some evidence to show that it addressed the existing relative gaps in the treatment of subclinical symptoms and distress by employing a transdiagnostic approach [42]. It should be noted that no negative clinical outcome was reported.

#### Personal Recovery Outcomes

Studies included in this review showed that online peer support interventions had overall a positive effect on personal recovery outcomes. Online peer support provides young people with mental health issues more opportunities to interact with similar users [52]. Shared experience and similar difficulties provided a sense of closeness between young individuals [10,19,23,45]. Consequently, young users were more likely to obtain better social support and relationship quality, feel the power of social integration, and improve social functioning [10,40]. Furthermore, connectedness was also helpful in reducing loneliness and isolation and improving life satisfaction [23,40,44,45]. In terms of other personal recovery outcomes, the intervention could help improve well-being, compassion, self-esteem, self-efficacy, sense of purpose, and hope [10,19,23,28,38,39,44]. Young people could be empowered to assist themselves, develop insight, and improve their ability to manage stress and solve problems through the acquisition of beneficial coping skills and strategies [19,38,42]. Subsequent online peer support interventions have been demonstrated to facilitate enhanced reappraisal, sustained perseverative thinking, and diminished perceived impact of mental health challenges [2,36]. Additionally, the increased engagement was identified as a significant benefit of online peer support, whether in reciprocal interactions or civic behaviors [10,36,38,42,46]. Meanwhile, online peer support with cost-effective nature has been proposed as a potential solution to the challenges posed by shortages of offline mental health care providers and support services [2,44].

Potential challenges of personal recovery outcomes are important aspects of online peer support that should not be ignored. Some studies acknowledged that the relatively limited evidence of effectiveness of online peer support is a potential challenge, especially in the context of the existing evidence for interventions delivered by professionals [40,42]. It was also suggested its effectiveness may be negatively affected by high attrition and lack of overall adherence, although these may also be challenges for other interventions [37,42]. No additional risks were identified in the remaining studies.

### Multidimensional Experiences of Online Peer Support

Overall, included studies reported mainly positive experiences, although mixed and negative experiences were also documented and require careful consideration. Reported benefits included improved social connectedness, reduced feelings of loneliness, elevated self-esteem, sense of purpose, empowerment, and civic engagement [10,19,23,38,43,44,46,47,49,50,52,57,59,60,63,64]. Online peer support offered young people a safe space to share experiences without censure and to have those experiences recognized [30,51], fostering resonance with others facing similar challenges [20,46]. Studies also reported an increased opportunity for self-disclosure [55], making it easier to discuss difficult topics and fostering a sense of belonging within a supportive community [38]. It was suggested that this sense of community arose from relatedness and friendship between young users [19,43,46,47,52,59,60,64,65] and it helped to reduce the experience of loneliness and social isolation [23,38,44,49,50,57,60,63,65]:

*I’m just trying to find a place where I’m feeling less alone with these feelings.*

[50]

*I felt much more comfortable on there than I did with many other apps because it felt very much like a community.*

[38]

Other reported benefits included acquiring new information and self-help, increased confidence to share and interact with others, and improving communication skills [10,43,44,47,51,59,60,64,65]. Young people were more likely to both seek and provide altruistic social and emotional support, which some felt was lacking in professional psychotherapy [47,52,60,61,65]:

*…I have attempted before but now I feel like they don’t treat my feelings seriously like if I’m not hurting or trying to take my life…nobody around me understands me. But on kooth, I feel like the community does. At least on here, there are people who can say that it isn’t just you Yano. You don’t have to go it alone. We take you seriously…*

[52]

Furthermore, some young individuals developed professional and academic aspirations through participation [19].

Positive experiences were linked to practical advantages such as overcoming geographical and time constraints, requiring only basic technical access to engage [38,49]. Several studies noted reduced stigma as a result of engagement [23,47,50,54]. Further, some participants described a shift in their perspective and letting go of preconceived ideas about coping, feeling supported despite isolation [20,57], and becoming more open to advice from peers [44,48,50-53].

*Having the reviews and the comments from peers who have utilized those different groups was...a huge thing that doesn’t exist anywhere.*

[53]

However, not all experiences were positive. Some young participants found that certain language use or stereotyping in lived experience stories could also intensify the stigmatizing impact [50]. Inaccurate or non–evidence-based information was a recurring concern [30]. Some users experienced a sense of invalidation, as well as normalization of their situations or symptoms through interactions, which may potentially discourage them from seeking help [38,50,51,65]. Conversely, some young people who provided support reported feelings of inadequacy, as well as fatigue, in relation to their role in supporting others [10,30,51,65]:

*Situations like sexual abuse and some more complicated situations that honestly I don’t know how to help with or I don’t even connect with because luckily I’ve never experienced. So for me it’s just like I don’t know how to show support so I prefer not to say anything. But then I feel bad because I see that they don’t have any replies and it’s a bit tricky because you don’t want that person to be without any support, but at the same time you don’t know how to support them or what to say or how to help really.*

[65]

In instances where constructive support and engagement were absent, adverse outcomes were observed, such as amplifying prevailing negative thought patterns [51,63,65]. Online peer communities appeared particularly risky in the context of self-injury, where toxic competition could foster harmful communication, worsen mental health, and normalize self-harm [50,51]:

*At first, you think this is great because they understand me and they get me, but also, they have the same issues as you… it can become that echo chamber or thoughts and feelings that just you can’t escape from…it kind of feels like a competition of who’s had it worse? Who cuts deeper? Who hurts themselves worse?*

[51]

Additional concerns included privacy breaches and cyberbullying, especially when digital technologies were misused by young individuals or when appropriate moderation was lacking [47]. Furthermore, some participants expressed a preference for more in-depth, personalized communication [59].

Beyond these inherent limitations, the design of online platforms or applications may also impede the advantages of peer support. The absence of user control over content selection was reported to potentially exacerbate mental health challenges [38]. Technical issues and poor integration with other apps were noted to reduce user satisfaction, with some platforms offering limited tools and instead directing users to features found in external applications [53]:

*I tried the search. I tried to look at different features that it had…in the first few times I tried it, it was kind of glitchy and I had to go back and restart.*

*I like to have everything sort of integrated into one application…just because it’s too much work to have to go and change apps, and you know, it’s more work and I’d be less likely to do it.*

Participants also described certain platforms as “dull,” “unintuitive,” or “clunky,” making them less appealing to young people with mental health concerns [23]. While safety considerations led some platforms to adopt strict moderation policies, this could contribute to delays in accessing counseling services, despite its easier online accessibility [44].

## Discussion

### Principal Findings

This scoping review synthesized findings from 38 studies on the effectiveness and experiences of online peer support for young people with mental health issues. Overall, largely positive effects were reported for both clinical and personal recovery outcomes, highlighting the great potential of online peer support alongside traditional services. However, findings for personal experiences were mixed.

In this review, online peer support was conducted in different countries, including the United Kingdom, the United States, Australia, China, Singapore, South Korea, Indonesia, and South Africa. The majority of online peer support, based on social media or specific mental health platforms, connects young users with others who have similar experiences of mental health challenges around the world and helps them develop new friendships in informal spaces, facilitating the expansion of the beneficiary population from any country and reducing geographical constraints, which is in accordance with the findings of a previous study [70]. At the same time, we found evidence that online peer support might decrease social barriers that hinder mental healthcare support seeking in particular stigmatization [71,72].

Regarding the association between effectiveness (or experiences) and different types of online peer support, our results reveal that young people reported limited effectiveness and more challenges using online peer support without moderation or lack of professional involvement. However, we did not find that other different characteristics (eg, asynchronicity/synchronicity, targeted general/specific mental health conditions) directly affect the benefits of online peer support, which makes it difficult to answer the question about effectiveness (or experience) across the different types of online peer support, but might have an impact on participation and interaction with help seekers and peer supporters [73]. Future empirical studies should focus on the specific effects of online peer support with varying characteristics on participants (eg, participation style) as well as how these factors further influence the effectiveness and positive experiences of online peer support.

### Online Peer Support and Traditional Services

Our results suggest that online peer support is effective across 2 domains: traditional clinical outcomes (eg, reduced mental health symptoms) and personal recovery outcomes, such as connectedness, hope, self-esteem, and empowerment, which may be absent in professional psychotherapy. Drawing on lived experience from peers is a core component of online peer support and has been found to engage young people who might otherwise not seek help [42]. Nevertheless, online peer support programs cannot replace mental health professionals [22,74]. Some young people prefer professional guidance for a tailored therapeutic approach [65].

The findings reported in this systematic scoping review are in line with some evidence of effectiveness of online peer support proposed by Ali et al [26] but also further identify the overall positive outcomes and extend the possible pathway combining online peer support and traditional mental health services together. The strengths of each approach are complementary. Online peer support offers resonance through shared experience and a low threshold for engagement, whereas traditional services provide professional mediation. Combining both may optimize outcomes for young people with mental health difficulties. The global shortage of youth mental health services is a significant barrier [75], and most online peer support programs have emerged from academic or commercial settings [46]. Our evidence for the effectiveness of online peer support further emphasizes the need for consideration to integrate online peer support into clinical care. It could be utilized at various stages of a patient journey [76], such as during waiting periods, alongside ongoing therapy, post-discharge, or preventatively. The current review specifically highlights the potential of integrating online peer support and traditional services.

### Online Peer Support and Online Platforms (Social Media)

Online peer support is delivered via dedicated mental health platforms (eg, Kooth, ReachOut) or social media (eg, TikTok, Instagram). Both have been demonstrated to yield positive effects for young people grappling with mental health challenges [52,53,77]. Mental health platforms support structured peer exchange, whereas social media enables user-generated content and informal peer support [70,78,79]. Both formats may be web-based or app-based.

Despite these benefits, current online peer support platforms are not fully tailored to young users’ preferences. Limited personalization, technical malfunctions, and suboptimal operational efficiency contribute to high dropout rates, with many users abandoning support before benefiting from it [23]. Other young people report that the constant exposure to others’ low moods causes them discomfort [29,30,38]. Potential solutions are to enhance content-filtering features, particularly on platform homepages, allowing users to block sensitive material and avoid unintentional exposure to adverse influences [38]. Further, the integration with other platforms or apps could improve engagement by allowing young users to switch between sources, apply helpful information, and provide support to peers across multiple platforms [53].

While social media is highly appealing to young people, it presents unique challenges. Social media platforms rarely focus specifically on mental health, exposing users to a wider range of information and interactions that may be difficult to critically assess. This may increase the risk of self-misdiagnosis or the reinforcement of maladaptive perceptions of mental illness [79,80]. Moderation could serve as a safeguard; however, the optimal role, scope, and effectiveness of moderators on social media remain under-researched and require further investigation.

Designing engaging, safe, and integrated digital mental health support platforms is therefore essential to maximize the benefits of online peer support. Until then, young people will likely continue to use social media for such mental health support. Our findings underscore that social media platforms require careful moderation and guidance to protect vulnerable young users while supporting positive peer interactions.

### Implications

Integrating traditional services with online peer support seems the ideal roadmap for future mental health care. Both online mental health peer support and traditional mental health services have benefits and drawbacks. Online peer support can play a valuable role in providing young people who are temporarily unable to access traditional services with reliable and compassionate strategies for coping with mental health issues. It can also support those who are under-informed or apprehensive about mental health challenges by reducing stigma and fostering positive attitudes toward seeking support and treatment. At the same time, traditional in-person services remain essential, particularly in establishing effective links with online resources to enhance care during therapy and following discharge.

Digital platforms also have the potential to enhance the benefits of online peer support. Interactive, safe, and integrated platforms may increase attractiveness and engagement while allowing users to select services that align with their personal needs. For instance, they may connect with peers for emotional support, access practical information from lived experiences to aid self-management, or simply find a safe space to express themselves. At the same time, online environments present complex challenges. Therefore, it is important that young individuals are informed about both the benefits and risks of different platforms so they can choose appropriate forms of support and avoid potential harms. Ideally, the development of online peer support interventions will foster collaboration with professionals and relevant platforms, contributing to more accessible and safer options for young people experiencing mental health difficulties.

### Limitations

This systematic scoping review has several limitations. First, although Levac et al [32] recommend stakeholder consultation as part of the review process, this was not a major component of the current review, which may limit its comprehensiveness. Second, although multiple databases were searched, the choice of search terms may have led to the exclusion of some relevant studies. To mitigate this, we conducted hand searches of key journals, relevant reviews, and seminal papers; however, only one additional eligible study was included. Finally, the absence of sufficient details makes it difficult to determine whether the included studies are independent evaluations or supported by platforms. Therefore, the effectiveness and other users’ positive experiences of online peer support should be considered with a degree of caution.

### Conclusions

This systematic scoping review presents evidence for the effectiveness of online peer support in 2 main categories—clinical recovery and personal recovery—and considered reports of experiences of online mental health peer support. The results highlighted the potential of integrating online peer support interventions into youth mental health care. The findings of the review increase our understanding of how various forms of online peer support for young people could promote mental health well-being, as well as personal development and encourage positive interaction, while also revealing challenges associated with moderation, privacy, and the platform itself. Future research should focus on the integration of online peer support, traditional services, and various digital platforms to better address young people’s mental health needs and further examine the effectiveness as well as experiences in practice to maximize online peer support benefits and reduce risks.

## Supplementary material

10.2196/83139 Multimedia Appendix 1 Inclusion and exclusion criteria.

10.2196/83139 Multimedia Appendix 2 Effectiveness of online peer support.

10.2196/83139 Multimedia Appendix 3 Experiences of online peer support.

10.2196/83139 Checklist 1 PRISMA-ScR checklist.

## Abbreviations

PRISMA-ScR: Preferred Reporting Items for Systematic Reviews and Meta-Analyses extension for Scoping Reviews
