# Supplementary material for: Effectiveness and Experiences of Online Mental Health Peer Support for Young People: Systematic Scoping Review
Source: JMIR Ment Health. 2026 Feb 25;13:e83139. doi: 10.2196/83139 (PMC12935419; doi:10.2196/83139)
Supplement: Multimedia Appendix 1 [file mental-v13-e83139-s001.docx]

**Appendix 1.** Inclusion and exclusion criteria.

| Parameters | Inclusion criteria | Exclusion criteria |
| --- | --- | --- |
| Population | - Young people, aged up to and including 25 (Mean age), engaging with online mental health peer support | - People using online peer support aged over 25 |
| Concept | - The online peer support should be primarily aimed at supporting mental health (not just as one component of online peer support for other issues) - Studies should be included online peer support intervention - Mental health support should be provided by someone with lived experience of using mental health services and/or of mental health challenges | - Young people using online peer support not primarily aimed at improving mental health - Online peer support for physical health difficulties or other irrelevant aspects - Content about offline peer support intervention - Online mental health support which is not peer support |
| Context | - Online mental health peer support in any virtual community across all sectors and all countries - Studies should be reported in peer-reviewed publication - Full text available in English | - Literature reviews (although these were referred to in the introduction) - Not peer reviewed - Not English language |
