# Supplementary material for: Effectiveness and Experiences of Online Mental Health Peer Support for Young People: Systematic Scoping Review
Source: JMIR Ment Health. 2026 Feb 25;13:e83139. doi: 10.2196/83139 (PMC12935419; doi:10.2196/83139)
Supplement: Multimedia Appendix 2 [file mental-v13-e83139-s002.docx]

**Appendix 2.** Effectiveness of online peer support.

| Author  (Year) | Country | Data Source | Number of Recruited Participants | Intervention | Clinical Outcomes | Personal Recovery Outcomes | Potential Challenges |
| --- | --- | --- | --- | --- | --- | --- | --- |
| Radovic et al.  (2022)[35] | Not reported | Two clinics | N=38 (randomised)  N=18 (allocated to peer support website) | Asynchronous, moderated (researchers) and specific | 1. Increased engagement with mental health treatment (61% at 6 weeks, 83% at 12 weeks)  2. Reduced negative feelings toward antidepressants | Not reported | Not reported |
| Morris, Schueller and Picard (2015)[36] | Not reported | Various universities, Internet websites (craigslist, research portals), and social media (Facebook, Twitter) | N=217 | Asynchronous, moderated (consumer and paid workers) and specific | (Cognitive-Behavioral Therapy-CBT as the primary therapeutic approach behind online peer support system)  Less depression | 1. Improved reappraisal  2. Increased perseverative thinking  3. Increased engagement | Not reported |
| Birrell et al. (2023)[37] | Australia | 12 independent and public  secondary schools | N=166 | Asynchronous and general | Reduced depressive symptoms | Not reported | 1. Lack of engagement  2. Lack of overall adherence |
| Ravaccia et al.  (2022)[38] | Not reported | Tellmi | Quantitative (876):  T1: N=398  T2: N=559  Both: N=81 | Asynchronous, moderated (trained and paid peer moderators) and general | Not reported | 1. Increased levels of patient activation for young females  2. Better mental health empowerment  3. Greater levels of engagement | Not reported |
| Kruzan et al. (2022)[39] | Not reported | TalkLife | N=131 | Asynchronous and specific | Reduced non-suicidal self-injury (NSSI) behaviour | Increased hope | Not reported |

**Appendix 2** (continued)

| Author  (Year) | Country | Data Source | Number of Recruited Participants | Intervention | Clinical Outcomes | Personal Recovery Outcomes | Potential Challenges |
| --- | --- | --- | --- | --- | --- | --- | --- |
| Ludwig et al.  (2020) [40] | The United States (North Carolina) | Three first-episode psychosis (FEP) clinics | N=26 | Asynchronous, moderated (professionals) and specific | (Integration in coordinated specialty care settings)  1. Reduced psychosis-related symptoms  2. Reduced negative emotions  3. Reduced depressive symptoms | 1. Reduced loneliness  2. Improved social integration and social functioning  3. Better perceived social support and relationship quality | Limited effectiveness |
| Arjadi et al. (2018) [41] | Indonesia | Community | N=159 | Asynchronous, moderated (trained lay counsellors) and specific | 1. Reducing depressive symptoms  2. Remission induction from a depressive episode | Not reported | Not reported |
| Bernecker et al.  (2020) [42] | The United States (Western Massachusetts region) | Several medium-sized towns | N=60 | Asynchronous and general | Improved treatment of subclinical symptoms and distressing | 1. Higher adherence  2. Improved ability to address stress and solve problems  3. Developed insight | 1. Not as powerful as those delivered by professionals  2. High attrition |
| Amon et al. (2022) [43] | Not reported | Phase 2 of Kids Helpline Circles | N=552 | Asynchronous, moderated (peer support experts) and general | 1. Reduction in depression  2. Reduced anxiety  3. Reduced stress  4. Reduction in general distress symptoms | Not reported | Not reported |
| Coote et al. (2024) [2] | The United Kingdom | Kooth | N=1520 | Asynchronous, moderated and general | 1. Decreased suicidal ideation  2. Decreased self-harm | Fewer perceived impact of difficulties | Not reported |

**Appendix 2** (continued)

| Author  (Year) | Country | Data Source | Number of Recruited Participants | Intervention | Clinical Outcomes | Personal Recovery Outcomes | Potential Challenges |
| --- | --- | --- | --- | --- | --- | --- | --- |
| Pavarini et al.  (2023) [10] | The United Kingdom | Social media | N=100 | Synchronous, moderated (peer support experts) and general | 1. Better mental wellbeing  2. Lower negative emotional symptoms | 1. Improved social support  2. Increased self-reported compassion  3. Connectedness  4. Increased engagement in civic behaviours  5. Greater self-efficacy | Not reported |
| Stevens et al.  (2022) [44] | Not reported | Kooth  (more than 1,500 children and young people log in to Kooth every day) | Quantitative: N=302 | Asynchronous, moderated (professionals) and general | 1. Reduced psychological distress  2. Reduced suicidal ideation  (peer support or combination of peer support and online counselling) | 1. Increased hope  2. Increased self-esteem  3. Reduced loneliness | Not reported |
| Mindel et al. (2022)[45] | Not reported | Kooth | Not reported | Synchronous and asynchronous, moderated (professionals) and general | Not reported | 1. Connectedness  2. Reduced isolation | Not reported |
| Alvarez-Jimenez et al.  (2024) [46] | Australia | 262 clinics | N=5702 | Asynchronous, moderated (trained and paid peer workers) and general | 1. Reduced depression  2. Reduced anxiety  3. Reduced psychological distress  4. Improved metal health wellbeing | Increased engagement (55% active) | Not reported |

**Appendix 2** (continued)

| Author  (Year) | Country | Data Source | Number of Recruited Participants | Intervention | Clinical Outcomes | Personal Recovery Outcomes | Potential Challenges |
| --- | --- | --- | --- | --- | --- | --- | --- |
| Van Meter and Agrawal (2024) [23] | The United States | Social media | N=202 | Asynchronous, moderated (researchers) and general | 1. Reduced depression  2. Reduced anxiety  3. Reduced suicidal ideation  4. Reduced non-suicidal self-injury (NSSI) | 1. Improved connectedness  2. Improved hope  3. Improved sleep quality  4. Improved life satisfaction | Not reported |
| Pavarini et al.  (2024) [19] | The United Kingdom | Social media  and schools | N=100 | Synchronous and general | Improved mental wellbeing | 1. Greater social connectedness  2. Improved coping skills  3. Increased sense of purpose  4. Increased self-esteem  5. Increased self-compassion | Not reported |
| Yeo et al. (2023) [28] | Singapore | A university | N=100 | Asynchronous, moderated (professionals) and general | Lower depressive and anxiety symptoms | 1. Enhanced selfhood  2. Enhanced compassion  3. Enhanced mindfulness | Not reported |
