# Supplementary material for: Effectiveness and Experiences of Online Mental Health Peer Support for Young People: Systematic Scoping Review
Source: JMIR Ment Health. 2026 Feb 25;13:e83139. doi: 10.2196/83139 (PMC12935419; doi:10.2196/83139)
Supplement: Multimedia Appendix 3 [file mental-v13-e83139-s003.docx]

**Appendix 3.** Experiences of online peer support.

| Author  (Year) | Country | Data Source | Number of Recruited Participants | Intervention | Positive Experiences | Negative Experiences |
| --- | --- | --- | --- | --- | --- | --- |
| Lavis and Winter (2020) [30] | Not reported | Twitter, Reddit, & Instagram | N=10,169 (original posts) and 36,934 (comments)  N=10  (interviews with self-harm content) | Asynchronous, moderated (self-surveillance) and specific | 1. Feeling listened to without judgment  2. Validation of painful, intimate and stigmatised experiences  3. Filling gaps in offline support | 1. Emotional burden of supporting others  2. Potential social contagion  3. Tension with clinical discourses |
| Aschbrenner et al.  (2019) [47] | The United States (Northeastern U.S.) | Four community mental health centres in one state | N=238  (121 from four community mental health centers and117 university students) | Asynchronous, moderated (professionals) and general | 1. Improved self-esteem  2. Perceived social support  3. Social capital growth  4. Improved communication skills  5. Greater confidence in social interaction and stronger social connectedness  6. Reduced stigma | 1. Privacy concerns  2. Risk of cyberbullying |
| Bennett et al.  (2022) [48] | Not reported | Childline (online peer support message boards) | Not reported | Asynchronous, moderated (professionals) and specific | Positive change in state or willingness to seek help | Not reported |
| Ravaccia et al.  (2022) [38] | Not reported | Tellmi | Qualitative:  N=84 (10 young users and  74 secondary students) | Asynchronous, moderated (trained and paid peer moderators) and general | 1. Making it easier to talk about difficult things  2. Being part of a supportive community  3. Providing new ways to help oneself  4. Feeling better  5. Feeling less alone  6. Easy usage | 1. Deterrence from usage  2. Resurfacing of distressing thoughts and feelings |

**Appendix 3** (continued)

| Author  (Year) | Country | Data Source | Number of Recruited Participants | Intervention | Positive Experiences | Negative Experiences |
| --- | --- | --- | --- | --- | --- | --- |
| O’dea and Campbell (2011) [49] | Australia  (Northern NSW) | A rural high school | N=74 | Asynchronous and general | 1. Providing pathways for isolated youth  2. Developing self-confidence  3. Breaking geographical limitations | Not being crucial for social contact |
| Winstone et al.  (2023) [50] | The United Kingdom | Another  experimental study | N=13 | Asynchronous and specific | 1. Motivating or inspiring to seek help  2. Empowerment to understand and share their own feelings and experiences  3. Reduced feelings of stigma  4. Reduced isolation | 1. Invalidation and deterrence from seeking help  2. Promotion of toxic competition and normalisation of self-harm  3. Stigmatising effect |
| Thorn et al. (2023) [51] | Australia | Organic, Instagram and workshop email list | N=20 | Not reported | 1. Access to helpful information, validation, and support  2. Encouragement of help seeking  3. Reinforcement of adaptive behaviour and achievements | 1. Ineffective online peer support  2. Emotional burden of supporting others  3. Normalisation and validation of self-harm  4. Worsening of self-harm and deterioration of general mental health |
| Amon et al. (2022) [43] | Not reported | Phase 2 of Kids Helpline Circles | N=552 | Asynchronous, moderated (peer support experts) and general | 1. Learning new coping skills  2. Feeling more socially connected  3. Empowering to tackle challenges | 1. Higher attrition  2. Lower adherence |
| Pavarini et al.  (2023) [10] | The United Kingdom | Social media | N=100 | Synchronous, moderated (peer support experts) and general | 1. Increased ability to support and connect with peers  2. Empowerment  3. Civic engagement  4. Self-care | Fatigue |

**Appendix 3** (continued)

| Author  (Year) | Country | Data Source | Number of Recruited Participants | Intervention | Positive Experiences | Negative Experiences |
| --- | --- | --- | --- | --- | --- | --- |
| Prescott, Hanley, and Ujhelyi (2017) [52] | Not reported | Kooth  (4400 posts within 2 years) | N=622 (initial posts), 3657 (initial posts and responses), 8 (moderator initial posts), and 113 (moderator posts and responses) | Asynchronous, moderated (professionals) and general | 1. Providing less loneliness, sense of normality and emotional support  2. Fostering motivation to share  3. Developing a sense of community and friendship | Not reported |
| Stevens et al.  (2022) [44] | Not reported | Kooth  (more than 1,500 children and young people log in to Kooth every day) | Qualitative:  N=10 | Asynchronous, moderated (professionals) and general | 1. Feeling more confident and more able to deal with relationships  2. Feeling less loneliness and a sense of fulfilment  3. Learning or developing self-help strategy  4. More motivation to seek help for mental health issues | 1. Upset from social comparison with negative experiences  2. Issues around waiting times |
| Wong et al. (2021) [53] | Not reported | Thought Spot | N=17 | Synchronous and asynchronous and general | 1. Improving user engagement  2. Increased motivation to access services | 1. Technical glitches  2. A lack of integration with other apps |
| Webb, Burns and Collin (2008) [54] | Not reported | Reach Out!  (over 6.5 million individual visits since 1998, with 230,000+ per month) | Not reported | Asynchronous, moderated (peer moderator, youth ambassadors and professionals) and general | 1. Improved mental health literacy  2. Stigma reduction  3. Increased help-seeking behaviour  (with the influence of CBT) | Not reported |
| Lerman et al.  (2017) [55] | Not reported | Facebook | All posts and replies within 6 active Facebook groups | Asynchronous and specific | Opportunity to safe disclosure | Limited effectiveness |
| Jin et al. (2023) [56] | China | NetEase Cloud Music (NCM) | N=13 | Asynchronous and general | Reducing psychological distress and maintain mental health | Not reported |

**Appendix 3** (continued)

| Author  (Year) | Country | Data Source | Number of Recruited Participants | Intervention | Positive Experiences | Negative Experiences |
| --- | --- | --- | --- | --- | --- | --- |
| Obuaku-Igwe  (2020) [57] | South Africa | A university | N=230 | Asynchronous and general | 1. Being willing to give up pre-conceived notions about life/things  2. Building a sense of solidarity to isolation  3. Supplement limited professional services | Not reported |
| Kim and Hong (2021) [58] | South Korea | Six universities | N=20 | Asynchronous and general | Not reported | 1. Lack of engagement  2. Emotional contagion an induced fear |
| Pokowitz et al.  (2023) [59] | The United States | Horyzons USA | N=20 | Asynchronous, moderated (peer support specialists and professionals) and general (most participants often had a diagnosis of schizophrenia) | 1. Fostering a sense of relatedness  2. Promoting confidence in interacting | 1. Limited range of communication  2. Lack of engagement |
| Prescott, Hanley and Gomez (2019) [60] | Not reported | Kooth | N=19 (participants) and 400 comments/responses | Synchronous and asynchronous, moderated (professionals) and general | 1. Feeling less alone and more connected to others  2. Learning new information  3. Changing moods | Not reported |
| Bickerstaff et al.  (2021) [61] | The United States | Universities, local mental health clinicians and the online university | N=40 (blog posts and corresponding comments written by 18 participants) | Asynchronous, moderated (professionals) and specific | 1. Providing hope  2. More altruistic interactions | Not reported |
| Gowen, Gruttadara and Markey (2012) [62] | Not reported | Social  networking websites | N=274 | General | 1. Communicating with other website users  2. Opportunity to help others | Not reported |

**Appendix 3** (continued)

| Author  (Year) | Country | Data Source | Number of Recruited Participants | Intervention | Positive Experiences | Negative Experiences |
| --- | --- | --- | --- | --- | --- | --- |
| Kruzan et al.  (2023) [63] | Not reported | Web | N=96 | Synchronous and asynchronous, moderated (administrative moderators) and specific | 1. Improvements in mood  2. Feeling less alone | 1. Worsening self-injury behaviours  2. Diminishing mood |
| Alvarez-Jimenez et al.  (2024) [46] | Australia | 262 clinics | N=5702 | Asynchronous, moderated (trained and paid peer workers) and general | 1. Higher satisfaction  2. Connecting with others experiencing similar challenges | Not reported |
| Van Meter and Agrawal (2024) [23] | The United States | Social media | N=202 | Asynchronous, moderated (researchers) and general | 1. Reduced isolation  2. Concern normalisation | Unappealing to young people |
| Carlisle et al.  (2024) [64] | Australia | Reach Out!  (more than 2 million Australian users annually) | N=1,000 (posts from 2018–2020) | Asynchronous, moderated (trained and paid staff, trained peer volunteers, and unpaid peer moderators) and general | 1. Creating a safe and trusted space for social connection  2. Providing opportunities for learning  3. Improved connection and belonging  4. Increased self-efficacy | Not reported |
| Pavarini et al.  (2024) [19] | The United Kingdom | Social media  and schools | N=100 | Synchronous and general | 1. Positive mental health  2. Higher sense of purpose  3. Better peer connectedness  4. Developing professional and academic aspirations | Not reported |

**Appendix 3** (continued)

| Author  (Year) | Country | Data Source | Number of Recruited Participants | Intervention | Positive Experiences | Negative Experiences |
| --- | --- | --- | --- | --- | --- | --- |
| Lau et al.  (2024) [20] | Not reported | TikTok  (with 1.5 billion monthly active users in 2023 and is expected  to reach 2 billion by the end of 2024) | N=100 (TikTok videos) | Asynchronous and general | Helping to shape positive attitudes | Negative engagement with the video |
| Cliffe et al. (2024) [65] | Not reported | Tellmi | N=11 | Asynchronous, moderated and general | 1. More altruistic support  2. Eliciting a sense of community  3. Learning skills  4. Feeling less alone and developing friendship | 1. Normalising situations or symptoms to prevent help-seeking  2. Pressure and responsibility to support  3. Exacerbating negative thoughts and feelings  4. Limited utility in resolving issues |
